# Sensitive Detection of Paraquat in Water Using Triangular Silver Nanoplates as SERS Substrates for Sustainable Agriculture and Water Resource Management

**DOI:** 10.3390/nano15231827

**Published:** 2025-12-03

**Authors:** Apinya Ketkong, Thana Sutthibutpong, Noppadon Nuntawong, Fueangfakan Chutrakulwong, Kheamrutai Thamaphat

**Affiliations:** 1 Green Synthesis and Application Laboratory, Applied Science and Engineering for Social Solution Research Unit, Department of Physics, Faculty of Science, King Mongkut’s University of Technology Thonburi, Bangkok 10140, Thailand; apinya.kk@mail.kmutt.ac.th; 2 Theoretical and Computational Physics (TCP) Group, Department of Physics, King Mongkut’s University of Technology Thonburi (KMUTT), Bangkok 10140, Thailand; thana.sut@kmutt.ac.th; 3 Center of Excellence in Theoretical and Computational Science (TaCS-CoE), Faculty of Science, King Mongkut’s University of Technology Thonburi (KMUTT), Bangkok 10140, Thailand; 4 National Science and Technology Development Agency (NSTDA), Pathum Thani 12120, Thailand; noppadon.nuntawong@nectec.or.th; 5 Division of Physics, Faculty of Science and Technology, Rajamangala University of Technology Krungthep, Bangkok 10120, Thailand; fueangfakan.c@mail.rmutk.ac.th

**Keywords:** triangular silver nanoplates, surface-enhanced Raman scattering, paraquat detection

## Abstract

This research focused on the synthesis of triangular silver nanoplates (TSNPs) with sharp corners using a photomediated seed growth method. The TSNPs produced had an average edge length of 27.2 ± 9.2 nm and a (110) crystalline plane structure. In terms of optical properties, the TSNPs displayed three key absorbance peaks at approximately 400 nm, 500 nm, and 660 nm, which correspond to out-of-plane dipolar resonance, in-plane quadrupolar resonance, and in-plane dipolar resonance, respectively. The prepared TSNP colloidal solutions served as surface-enhanced Raman spectroscopy (SERS)-active materials for detecting paraquat residue in aqueous samples. We optimized the mixing time of the liquid SERS with the sample, maintaining a 1:1 volume ratio. The findings showed a remarkable enhancement of the Raman signal with 10 min mixing time using laser excitation at a wavelength of 785 nm. This study achieved the development of novel SERS-active substrates capable of detecting pesticides with excellent accuracy, sensitivity, and reproducibility for both qualitative and quantitative analysis in tap water, river water, drinking water, and cannabis water. Additionally, it paved the way for using the SERS technique as a promising approach in the areas of food safety and environmental monitoring, especially in the organic farming field.

## 1. Introduction

The global population is projected to reach nearly 10 billion by 2050, placing unprecedented demands on agricultural systems worldwide [1,2]. Meeting the resulting demand for food requires substantial production increases, with the agriculture market already showing strong growth—from USD 13,272.75 billion in 2023 to an estimated USD 14,356.23 billion in 2024 (CAGR 8.2%). Overall, global food production must rise by approximately 70%, a challenge further intensified by climate change, which is projected to worsen resource shortages and disrupt planting cycles [2,3]. In response to increasing agricultural pressures, the legacy and continued use of synthetic chemicals—such as fertilizers and pesticides—has played a significant role in enhancing crop yields in regions where their application remains legal [4,5]. Although effective in the short term, these chemicals accelerate soil degradation and promote the emergence of herbicide-resistant weeds. Consequently, the use of broad-spectrum herbicides, including paraquat (PQ), glyphosate, atrazine, and glufosinate, remains widespread for controlling aggressive weed populations [6,7]. PQ is particularly favored due to its rapid disruption of photosynthetic electron transport [6]; however, it poses serious ecological and health risks, including central nervous system damage and potential associations with neurodegenerative diseases such as Parkinson’s disease [8]. As a result, its use has become increasingly regulated or banned across numerous regions, beginning in Norway, extending to several other European countries, and ultimately resulting in a European Union–-wide ban in 2007, although it continues to be used under strict regulatory oversight in China and the United States [9,10]. Despite these restrictions, PQ use persists in areas with weak enforcement, where improper or unsafe application practices are commonly observed, and historical applications have left long-lasting residues that strongly bind to organic-rich soils [5]. Moreover, the poor solubility and environmental persistence of PQ allow these residues to leach into natural water bodies, contaminating rivers, canals, and reservoirs that supply domestic consumption, agricultural irrigation, and food and beverage production. These concerns over chemical contamination have contributed to a 26.6% increase in organically farmed land between 2021 and 2022, as both farmers and consumers increasingly seek safer, chemical-free alternatives [11].

As widespread water contamination continues to raise public and regulatory concern, key agencies such as the U.S. Environmental Protection Agency (EPA) have established permissible PQ levels in drinking water at 30 mg/L, or 1.2 × 10^−7^ M, which is considered safe for human and animal health [12,13]. Traditional detection methods, including gas chromatography–mass spectrometry (GC-MS), liquid chromatography–mass spectrometry (LC-MS), and high-performance liquid chromatography (HPLC), are widely recognized for their precision, reliability, and ability to accurately quantify trace contaminants [14]. However, these conventional methods often fail to detect the ultra-low PQ concentrations required to meet organic farming standards [15,16], while complex environmental matrices can further reduce analytical accuracy [17], and their lengthy processing times may delay critical decision-making in situations requiring rapid assessment. Given these limitations, innovative detection methods offering both high sensitivity and rapid analysis are urgently needed. Surface-enhanced Raman spectroscopy (SERS), which employs metallic nanoparticles to amplify Raman signals through localized surface plasmon resonance (LSPR), provides a highly promising solution capable of detecting ultra-trace concentrations. Its compatibility with both bench-top and portable Raman spectrometers makes SERS well suited for field applications [4,5,13,18,19,20,21,22,23].

Based on the literature, SERS substrates are generally classified into two types: solid and liquid formats [24]. Solid substrates provide strong signal enhancement due to the ordered arrangement of metal nanostructures that generate abundant hotspots [20,25,26,27]. However, these fabrication methods, along with storage to prevent oxidation, specialized equipment, long production times, and high costs, are typically single-use and prone to contamination if reused. In contrast, liquid SERS substrates are more cost-effective, easily synthesized, and producible in large batches, allowing unlimited sample measurements. Although they may be sensitive to organic compounds in environmental matrices [20], their suitability for repeated measurements and diverse sample types enables robust statistical validation. The key factors in the colloidal SERS substrate synthesis include the choice of noble metal for SERS application; silver (Ag) is often preferred over gold (Au) and copper (Cu) because of its interband transition in the ultraviolet region, allowing more efficient scattering of visible and near-infrared monochromatic light and thereby enhancing the LSPR response. However, the formation of “hotspots”—regions of intense LSPR arising from the distribution of surface plasmons of various geometries—induces Coulombic interactions [5,28,29], and these hotspots are also enhanced by nanoparticle aggregation [21,28,30]. In addition, surface free energy of AgNPs promotes stronger adsorption of analyte molecules onto the surface [18,19,22,23,31,32]. Several studies have employed plasmonic colloidal nanoparticles of varying sizes and shapes for pesticide detection, such as spherical silver nanoparticles, silver nano-snowflakes, dog bone-shaped gold nanoparticles, silver nanostars, spherical gold nanoparticles, and silver triangular nanoplates [21,28,29,30,33,34]. The lowest PQ concentration detectable in previous studies was reported to be 10^−9^ M. Based on the studies described above, TSNPs are an attractive choice because their well-defined sharp tips and edges give rise to three distinct LSPR modes while also conferring greater mechanical robustness compared to similar structures. Their single-crystalline structure ensures the formation of stable hotspots, and the sharp tips on the (110) plane provide high surface free energy that promotes the adsorption of PQ molecules, eliminating the need for additional surface modification agents required in other studies [4,18,23,35]. Additionally, TSNPs can be synthesized with precise size control and high uniformity via photoirradiation, yielding plasmon resonances with high specificity. However, to date, no studies have investigated the direct detection of PQ residues by mixing TSNP colloidal SERS with contaminated water without sample extraction or requiring catalysts or surface modifiers and allowing the mixture to remain in its liquid form before being drop-cast for Raman analysis.

In this study, TSNP colloids were synthesized via a photoirradiation method for the detection of PQ in water using a simple procedure. The TSNPs were mixed with water samples at a 1:1 ratio in their liquid form, and electrostatic interactions enabled PQ molecules to adsorb uniformly onto the TSNPs surface without the need for catalysts or surface modifiers, distinguishing this work from previous studies. The mixture was then drop-cast onto an aluminum foil-coated glass slide, enhancing Raman signal amplification. This approach allows the detection of PQ at unprecedentedly low concentrations. The study also investigated the optimal mixing time of TSNP colloids and PQ and evaluated the method’s detection capability, limit of detection (LOD), limit of quantitation (LOQ), and overall performance through analysis of the signal-to-noise ratio (SNR), enhancement factor (EF), and relative standard deviation (RSD), which indicate Raman signal amplification, stability, and reproducibility. Furthermore, the accuracy of this method was validated by comparing PQ measurements in natural water samples with standard LC-MS analysis using the percentage of recovery, providing guidance for potential real-world applications.

## 2. Materials and Methods

### 2.1. Materials

The chemical substances were obtained from the following suppliers: Silver nitrate (AgNO_3_) from POCH (Gliwice, Poland), trisodium citrate (Na_3_C_6_H_5_O_7_) from Ajax Finechem (Cherrybrook, NSW, Australia), sodium borohydride (NaBH_4_) from QReC (Asia) Sdn. Bhd. (Kuala Lumpur, Malaysia), paraquat dichloride (PQ) from Dr. Ehrenstorfer (Augsburg, Germany), and deionized (DI) water from Green Synthesis and Application Laboratory (Bangkok, Thailand).

### 2.2. Synthesis of Triangular Silver Nanoplates (TSNPs)

**Figure 1:**
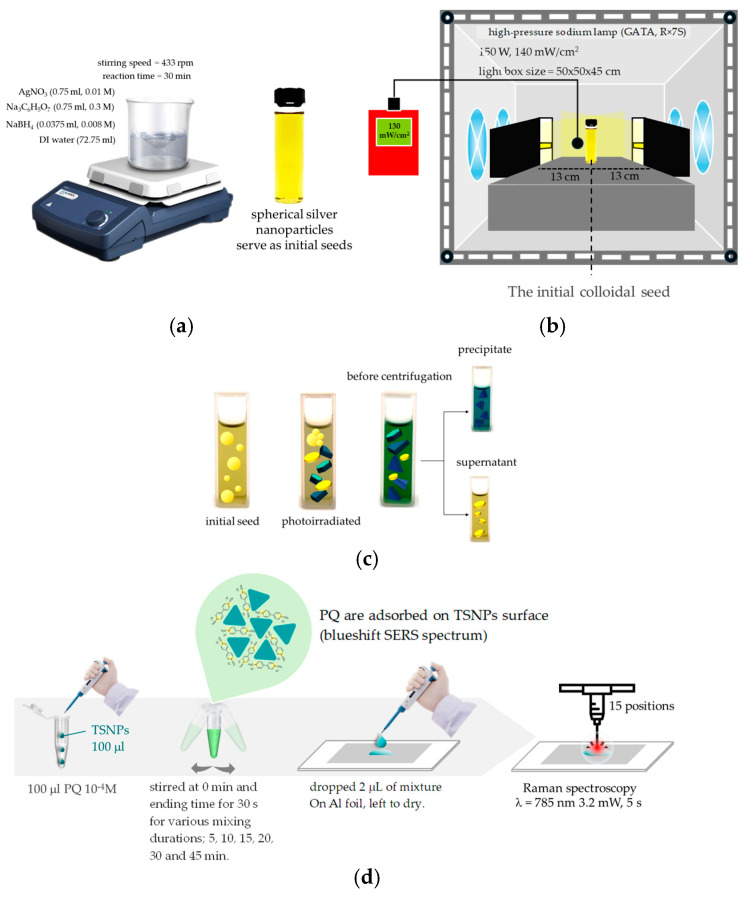
(**a**) Synthesis procedure of seed colloids. (**b**) Synthesis of TSNPs from seed colloid using photomediated seed growth method. (**c**) Collection of TSNPs from silver nanoparticle colloid using centrifugation. (**d**) Schematic of SERS measurement of PQ using TSNP colloid.

TSNPs were synthesized based on a previously reported method [36]. The synthesis of the TSNP colloid required the preparation of spherical silver nanoparticles (AgNSs) as initial seeds through a chemical reduction process. In brief, 72.75 mL of DI water was mixed with 0.75 mL of 0.01 M AgNO_3_ aqueous solution and 0.75 mL of 0.3 M Na_3_C_6_H_5_O_7_ aqueous solution sequentially. The mixture was stirred at 433 rpm for 30 min. After this period, 0.0375 mL of 0.008 M NaBH_4_ aqueous solution was added dropwise, and stirring was continued for an additional 2 min until the AgNS initial seeds were formed. Finally, the colloid was stored in a refrigerator and protected from light at 4 °C for 12 h. Then, 20 mL of the seed colloid was taken out of the refrigerator and allowed to acclimate to room temperature (25 °C) in a dark environment for approximately 2 h. Subsequently, the seed colloid was irradiated using light from high-pressure sodium lamps (FL, HPS-T 150W R×7S) (Luna, SC735, Bangkok, Thailand) for 1 h with 130 mW/cm^2^, with the containers positioned 13 cm away from the lamp surface on both sides (Figure 1a,b). After the light exposure, the TSNPs were distinguished from the AgNPs through centrifugation. Then, 1.5 mL of AgNPs was loaded into each of 12 microcentrifuge tubes and centrifuged at 15,000 rpm for 15 min using a high-speed tabletop microcentrifuge (UGAIYA, Hirakata, Osaka, Japan). A total of 1.4 mL of the supernatant was discarded, and only 0.1 mL of the TSNP pellets was kept. The TSNP pellets were loaded into a new container and diluted with deionized water to achieve a consistent absorbance wavelength range before using (Figure 1c).

### 2.3. Characterization of TSNPs

The light absorption of TSNPs was measured using UV-visible spectrophotometry (Avantes, AvaSpec-EDU, Bangkok, Thailand) within the spectral range of 350 to 800 nm. A consistent volume of 3 mL of the colloid was used for each measurement, with DI water serving as the blank. Transmission electron microscopy (TEM) (HT7800, Hitachi High-Tech Corporation, Tokyo, Japan) and electron diffraction (JEM-2100, JEOL Ltd., Tokyo, Japan) were utilized to assess the size, shape, crystal structure, and particle aggregation of the TSNP colloid when mixed with PQ for various mixing times. Samples were prepared by depositing 5 µL of the TSNP colloid onto a 300-mesh copper grid and allowing it to dry for at least 12 h in a desiccator, protected from light, at room temperature (25 °C).

### 2.4. SERS Measurement of PQ

#### 2.4.1. Preparation of Standard Solutions in Water Samples

Different concentrations (10^−4^, 10^−6^, 10^−8^, 10^−10^, 10^−12^, 10^−13^, 5 × 10^−14^, 5 × 10^−16^, and 10^−16^ M) of PQ standard were prepared. DI water was used as the solvent for the PQ standard solutions. Tap water (Suphanburi, Thailand), drinking water (Crystal, Sermsuk public company limited, Bangkok, Thailand), river water (Chao Phraya River, Bangkok, Thailand), and cannabis water (DEK420, Yan Wal Yun Company Limited, Bangkok, Thailand) were left to settle, allowing sediment to form. Subsequently, only the clear liquid from the upper layer was used for testing by spiking different amounts of PQ to create various concentrations of PQ samples.

#### 2.4.2. Characteristics of Raman Spectrum of PQ

To study the characteristic Raman signals of PQ using TSNP colloidal SERS-active substrate, a standard PQ solution (99% purity by mass) was prepared by dissolution in DI water to obtain a concentration of 10^−4^ M. Then, 0.1 mL of this 10^−4^ M PQ solution was mixed with 0.1 mL of the TSNP colloid and shaken for 30 s. After a 10 min incubation period, the mixture was shaken again for 30 s. A 0.002 mL aliquot of the mixture was then deposited onto aluminum foil affixed to a glass slide and allowed to dry. The sample was analyzed using a Raman spectrometer (Renishaw plc., inVia, Wotton-under-Edge, Glos, UK) with a 785 nm laser source. The laser beam (3.2 mW power) was focused with 50× magnification. The laser was focused for 5 s, and each measurement was averaged over 2 scans. The resolution range of 800–1700 cm^−1^ typically refers to a specific region. This data was compared with the Raman signals of a 10^−4^ M PQ solution that was not mixed with a TSNP colloid.

#### 2.4.3. Optimizing the Mixing Time of TSNP Colloids with PQ Standard Solution

To determine the optimal mixing time of TSNP colloids and PQ standard solution, 0.1 mL of a 10^−4^ M PQ standard solution was combined with 0.1 mL of the TSNP colloid and shaken for 30 s. The mixture was then allowed to rest for 5 min before shaking again for 30 s. A 0.002 mL portion of the mixture was then applied to aluminum foil attached to a glass slide and allowed to dry. Raman spectroscopy was performed using a Renishaw inVia spectrometer with a 785 nm laser with 3.2 mW power and 50× magnification, with a laser exposure time of 5 s and 2 scans averaged. The resolution range of 800–1700 cm^−1^ typically refers to a specific region. The experiment was repeated with mixing durations of 10, 15, 20, 30, and 45 min, and data were collected from 30 positions (Figure 1d). The Raman spectra were evaluated for signal enhancement based on the signal-to-noise ratio (SNR), enhancement factor (EF), and the likelihood of detecting the primary peak at 1651 cm^−1^.

#### 2.4.4. Performance of TSNP Colloidal SERS

PQ standard solutions were prepared at concentrations of 10^−4^, 10^−6^, 10^−8^, 10^−10^, 10^−12^, 10^−13^, 5 × 10^−14^, 5 × 10^−16^, and 10^−16^ M. A total of 0.1 mL of each PQ solution was mixed with 0.1 mL of the TSNP colloid, and the mixture was shaken for 30 s. The mixture was allowed to rest for the optimal mixing time determined previously then shaken again for 30 s. Afterward, 0.002 mL of the mixture was pipetted onto aluminum foil attached to a glass slide and left to dry. The dried sample was analyzed using a Raman spectrometer with a 785 nm laser with 3.2 mW power and 50× magnification. The laser was focused for 5 s per measurement. The resolution range of 800–1700 cm^−1^ typically refers to a specific region. This process was repeated for each PQ concentration. The average intensity (measured at 20 positions) of the Raman peak at 1651 cm^−1^ was plotted against the PQ concentration using a logarithmic function. The spectra were evaluated for Raman signal enhancement based on the SNR, EF, and the probability of detecting the primary peak at 1651 cm^−1^.

### 2.5. Simulation and Application of TSNP Colloidal SERS for Environmental PQ Detection

For environmental detection of PQ residues, commercial-grade PQ was added to water samples from various sources—river water, ground water, tap water, drinking water, and cannabis-infused water—to achieve a final concentration of 10^−7^ M (25.7 µg/L). The water samples were then analyzed using TSNP colloidal SERS under the previously optimized conditions. A 1:1 volume ratio of the spiked sample and TSNP colloid was mixed, shaken for the established optimal mixing time, and allowed to rest. Afterward, 0.002 mL of the mixture was transferred onto aluminum foil mounted on a glass slide and dried. Raman spectroscopy was conducted using a Renishaw inVia spectrometer with a 785 nm laser with 3.2 mW power, 50× magnification, and a 5 s exposure time. The resolution range of 800–1700 cm^−1^ typically refers to a specific region. The data obtained were compared to the percentage of recovery from standard liquid chromatography–mass spectrometry (LC-MS) to validate the accuracy and reliability of the SERS method.

## 3. Results

### 3.1. Structural Formation and Morphological Properties of TSNPs

**Figure 2:**
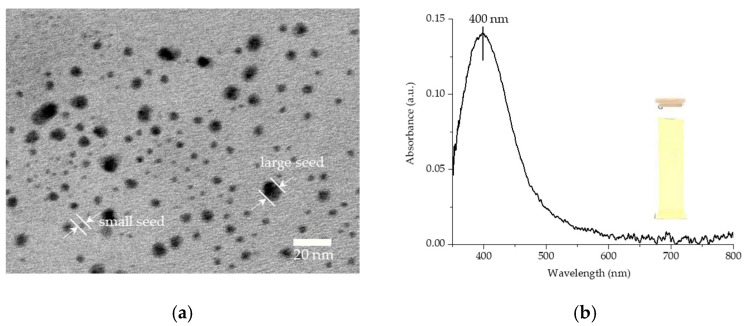
(**a**) TEM image. (**b**) UV-vis absorption spectrum of the as-prepared spherical Ag nanoseeds.

Spherical Ag nanoseeds were synthesized by reducing AgNO_3_ with NaBH_4_ in the presence of trisodium citrate. The reaction mechanism begins when AgNO_3_ is dissolved in DI water, dissociating to release Ag^+^ ions, which then accept electrons from BH_4_^−^ ions dissociated from NaBH_4_. This leads to the formation of solid silver (Ag^0^), which can be expressed as Equation (1) [37], which is capped by C_6_H_5_O_7_^3−^ ions dissociated from Na_3_C_6_H_5_O_7_ [33]. Ultimately, two seed sizes were obtained: small seeds measuring 3.5 ± 0.1 nm and large seeds measuring 7.6 ± 0.2 nm, as shown in the TEM image (Figure 2a). During synthesis of the seeds, the maximum absorption wavelength of the particles appears at 400 nm, which falls within the blue light region, leaving only yellow light transmitted through, resulting in a yellow, transparent colloid (Figure 2b).

(1) AgNO3+NaBH4→Na3C6H5O7Ag+12H2+12B2H6+NaNO3

**Figure 3:**
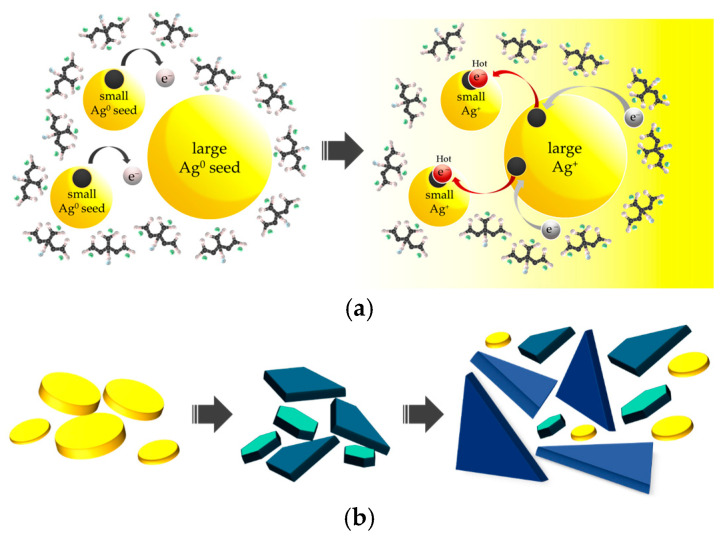
(**a**) The schematic of redox reaction occurring on the Ag nanoseed surface and (**b**) the transformation of spherical Ag nanoseeds to triangular Ag nanoplates under photoirradiation.

As shown in Figure 3a, under oxygen-rich colloidal conditions, smaller AgNPs are more prone to electron loss, forming Ag^+^ ions due to their lower redox potentials. When the colloid is irradiated at a wavelength of 589 nm, surface electrons of the larger seed particles become photoexcited, generating hot electrons, which are then transferred to the unoccupied orbitals of the pre-formed small Ag^+^ ions. Subsequently, the hot holes created on the large seeds are filled through the decarboxylation of citrate ions. Initially, the transformation of isotropic silver seeds into anisotropic shapes progresses slowly. However, as particle size increases, plasmon excitation becomes more pronounced, enabling more efficient energy absorption. This dipole plasmon excitation promotes a face-selective reduction of silver cations, specifically on the (111) twin planes, leading to the formation of TSNPs consistent with an Ostwald ripening process (Figure 3b) [36,38,39].

**Figure 4:**
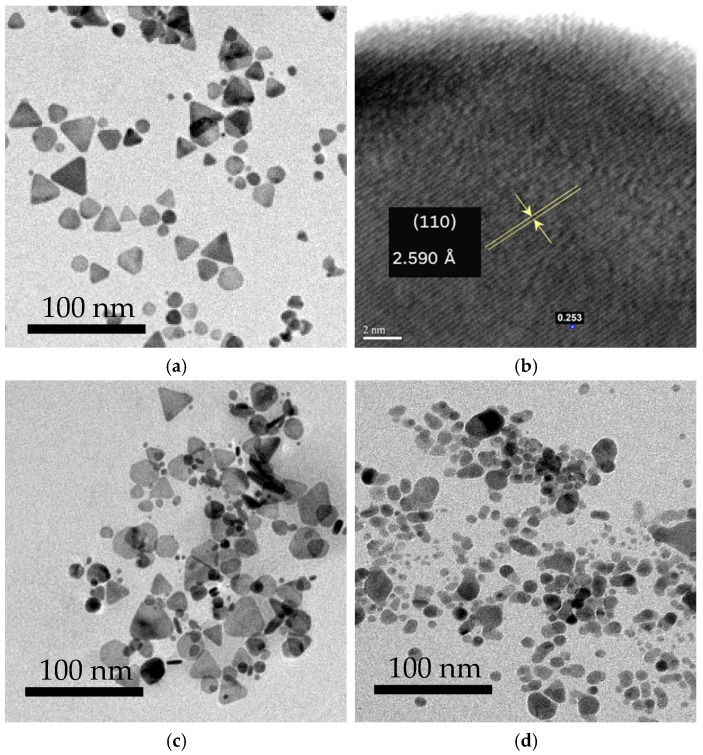
(**a**) HR-TEM and (**b**) TEM images of the synthesized TSNPs after separation. (**c**) AgNPs before and (**d**) AgNSs after separation.

**Figure 5:**
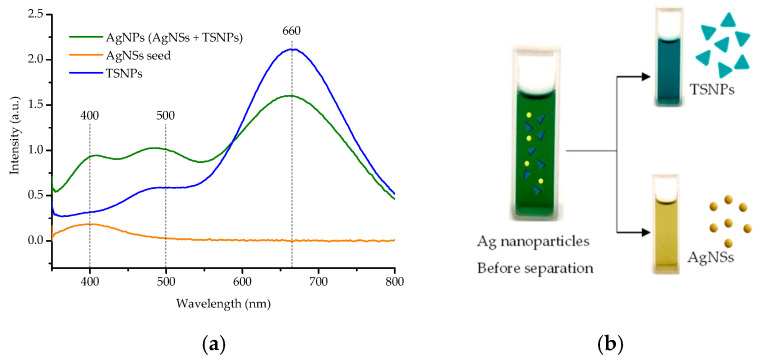
(**a**) UV-vis spectra of AgNS seeds, AgNPs, TSNPs, and AgNSs. (**b**) The colloidal color observed following the separation of particle morphologies.

The TEM images reveal that the synthesized TSNPs have an average edge length of 27.2 ± 9.2 nm (Figure 4a). Furthermore, the high-resolution TEM (HR-TEM) images confirm the TSNPs’ structure by showing an interplanar spacing of 2.590 Å, which corresponds to the (110) crystalline plane of the TSNPs (Figure 4b) [39]. In addition, the UV-Vis absorbance spectra of the AgNP colloid before particle separation display three distinct absorbance peaks at 400, 500, and 660 nm. These peaks are associated with the oscillations of the silver nanoparticles, which comprise both TSNPs and Ag nanospheres (AgNSs) in the form of out-of-plane dipolar resonance, in-plane quadrupolar resonance, and in-plane dipolar resonance, respectively (Figure 5a) [40,41]. However, after the particle separation process, approximately 43% of the total particles, which correspond to the TSNPs, remain available for further use (Figure 4a), displaying a single surface plasmon resonance (SPR) band at 660 nm. Consequently, this selective presence of TSNPs results in the clear blue appearance of the colloid (Figure 5b).

### 3.2. PQ Detection Using the TSNP Colloidal SERS

#### 3.2.1. Characteristics of the Raman Signal of PQ

**Figure 6:**
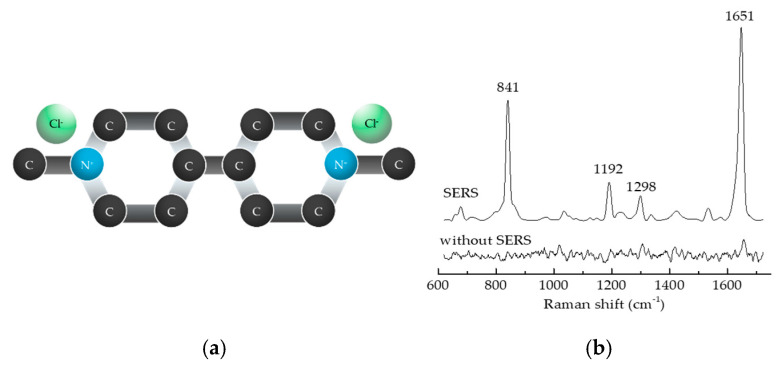
(**a**) PQ molecule. (**b**) Raman spectra of PQ molecules using and without TSNP colloidal SERS.

**Figure 7:**
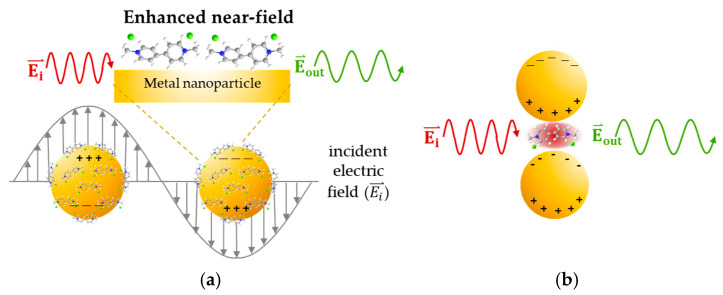
(**a**) The surface plasmon resonance phenomena. (**b**) The hotspot area of metal nanoparticle dimmer structure due to incident polarization parallel to the interparticle axis.

Figure 6a,b shows the comparison of Raman signal measurements for a 10^−4^ M PQ standard solution, both without the addition of TSNPs (non-SERS) and with TSNPs (SERS), revealing that the Raman signals of PQ (Table 1) exhibit very low peak intensities in the absence of SERS. However, when TSNPs are employed for SERS (Figure 7), the Raman signals become significantly more prominent. To compare the signal enhancement capability of TSNPs, the intensity of the main Raman signal at 1655 cm^−1^ is considered. However, when the N molecule of PQ adsorbs onto the surface of TSNPs, the main Raman signal shifts slightly to 1651 cm^−1^ [4,42].

#### 3.2.2. Optimal Mixing Duration for PQ Standard Solution and TSNPs

Three key factors are considered when assessing the suitability of the conditions:

• The most widely accepted definition of signal-to-noise ratio (SNR) is the ratio of the average peak height above the baseline to the standard deviation of the peak height. Another way to define the SNR is as the peak height above the baseline compared to the baseline noise or as the root mean square (RMS) value of a flat region in the spectrum [43]. Therefore, in this research, the SNR calculation was performed using the equation shown in Equation (2).

(2) $$signal-to-noise\;ratio\;\left(SNR\right)=\frac{primary\;peak\;intensity\;@1651\;{cm}^{-1}\;}{primary\;peak\;intensity\;@900\;{cm}^{-1}}$$

• The enhancement factor (EF) is the ratio of the SERS signal to the Raman signal that would be obtained for the same molecule under the same conditions. It indicates the amplification of the Raman signal due to the presence of nanostructured metallic surfaces, with the EF typically ranging from 10^4^ to 10^6^ [44]. Therefore, in this research, the EF calculation was performed using the equation presented in Equation (3).

(3) $$enhancement\;factor\;\left(EF\right)=\frac{primary\;peak\;intensity\;of\;SERS\;spectra\;or\;I_{SERS}}{primary\;peak\;intensity\;of\;Raman\;spectra\;or\;I_{normal\;Raman}}\times \frac{concentration\;of\;PQ\;or\;N_{normal\;Raman}}{concentration\;of\;PQ\;or\;N_{SERS}}$$

• The probability of the primary peak appearance in the detection area is represented as a red mapping grid with varying color intensities. The brightest red regions indicate the highest Raman signal intensity, which gradually decreases with darker shades of red, eventually turning to black, indicating the absence of the primary peak.

**Figure 8:**
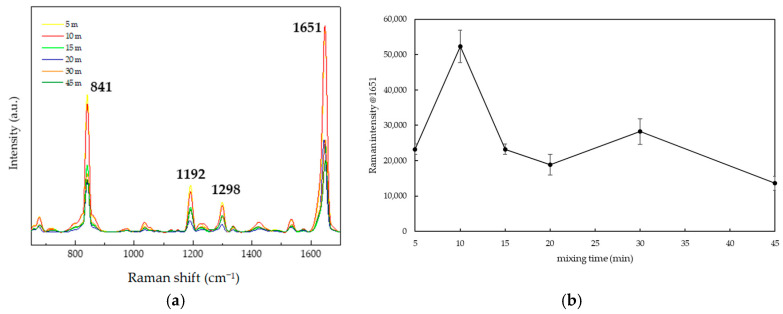
(**a**) Average SERS spectra. (**b**) Average primary peak intensity at 1651 cm^−1^ in detection of 10^−4^ M PQ mixed with TSNP colloids for six different mixing durations.

**Figure 9:**
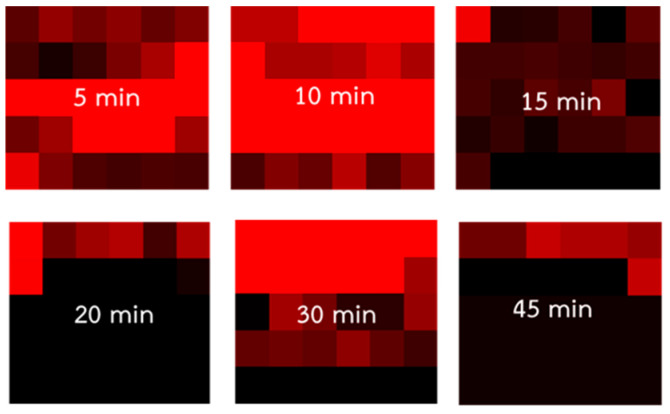
Probability mapping of primary peak appearance in detection area of 10^−4^ M PQ mixed with TSNP colloids for six different mixing durations.

**Figure 10:**
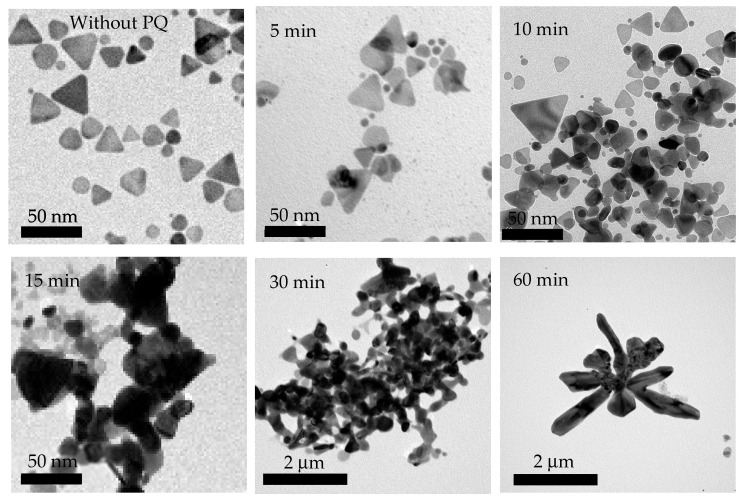
TEM images of TSNP aggregation induced by the combination with 10^−4^ M PQ for various mixing durations.

After considering three factors, a mixing time of 10 min was found to be the most suitable for practical applications, which is supported by the following reasons. First, the 1651 cm^−1^ primary peak of the SERS spectrum and average intensity of 10^−4^ M PQ solution with a 10 min mixing time is highest, as shown in Figure 8a,b. When examining the SNR of the Raman signal using SERS, it was found that the 10 min SNR value of 255.84 was higher than that of the non-SERS signal and the highest among all mixing times. Second, considering the EF, which should ideally be at least 10–100 times greater, the mixing time of 10 min resulted in a 10 min EF value of 2518.19, the highest among all mixing times. The primary peak intensity, SNR, and EF are summarized as shown in Table 2. Third, the mapping of the probability of detecting the Raman signal at 1651 cm^−1^ revealed that the 10 min mixing time produced a red area covering more than 56% (approximately 17/30 cells), the largest among all mixing times, as illustrated in Figure 9. Furthermore, additional TEM images of the TSNP colloid mixed with PQ at different times show that the TSNPs tend to aggregate due to the positively charged nitrogen on the PQ molecule, while the surfaces of the TSNPs are covered with a negative charge. This ion-pair interaction or strong affinity may cause the TSNPs to lose electrons densely located at the tips, resulting in a more rounded appearance. However, the interparticle distance remains suitable for maximizing Raman signal enhancement. Furthermore, at lower PQ concentrations, the reduction-induced corner truncation is proportionally diminished, allowing better preservation of the sharp edges, which are essential for generating intense LSPR. By 15–60 min of mixing, the TSNPs form larger cluster aggregates, making them unsuitable for further SERS applications, as shown in Figure 10.

#### 3.2.3. Performance of TSNP Colloidal SERS

**Figure 11:**
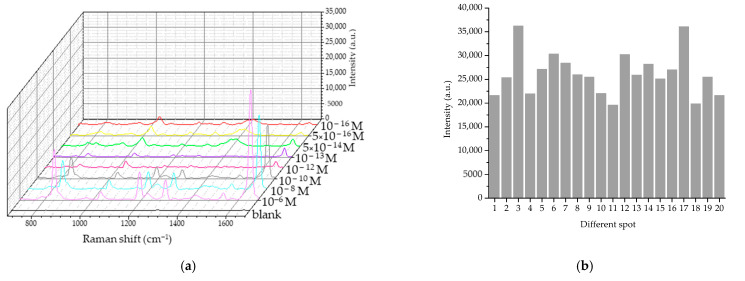
(**a**) Average SERS spectra of 10^−4^–10^−16^ M PQ detection using TSNP colloidal SERS and (**b**) bar chart of 20 replicate measurements of PQ aqueous solution using TSNP colloidal SERS.

**Figure 12:**
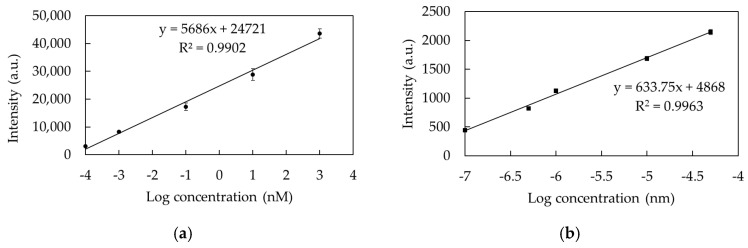
Calibration curve of PQ aqueous solution detection using TSNP colloidal SERS at concentrations of (**a**) 10^−6^–10^−13^ M and (**b**) 10^−14^–10^−16^ M.

In this study, the performed TSNP colloidal SERS was able to detect PQ standard solutions at concentrations as low as 10^−6^ M, which is the lowest concentration that could not be detected using conventional Raman spectroscopy under the same measurement conditions. Furthermore, when measuring PQ solutions at progressively lower concentrations, it was found that the TSNP colloidal SERS could detect concentrations as low as 10^−16^ M, a sensitivity surpassing that of previous studies. Although the ability to detect PQ concentrations in samples should be sufficient at 10^−7^ M, as per the EPA standard, the application of this method in organic farming requires the ability to detect extremely low concentrations of PQ, ideally approaching zero. Table 3 corresponds to the average SERS spectra shown in Figure 11a, presenting the primary peak intensity and SNR values for the detection of PQ at concentrations ranging from 10^−6^ to 10^−16^ M. The SNR results confirm that the Raman signals detected at each concentration are clearly attributable to the analyte and can be clearly distinguished from the background noise, even at extremely low concentrations. According to Equations (4) and (5), the limit of detection (LOD) and limit of quantitation (LOQ) [29] were validated at 4.62 × 10^−17^ M and 6.46 × 10^−17^ M with a Raman intensity of no less than 219 and 310, respectively, and the calibration curves are shown in Figure 12a,b. Additionally, the lifetime of PQ in water at a concentration of 10^−7^ M was selected to calculate the percentage of relative standard deviation (%RSD) using Equation (6) based on 20 spot measurements. The corresponding bar chart is displayed in Figure 11b. The %RSD value of 3.95% demonstrates high stability and reproducibility of the TSNP colloidal SERS detection method because a satisfactory RSD value is typically no more than 20% [30].

(4) $$LOD=I_{blank\;}+{3SD}_{blank}$$

(5) $$LOQ=I_{blank\;}+{10SD}_{blank}$$

where $I_{blank\;}$ refers to the average intensity of the primary peak at 1651 cm^−1^ of the blank and ${SD}_{blank}$ refers to the standard deviation of primary peak intensity with 20 replicate measurements of the blank.

(6) $$\%RSD\;=\;SD/\bar{x}\times 100\%$$

where *SD* refers to the standard deviation of primary peak intensity and $\bar{x}$ refers to the average intensity of the primary peak.

Table 4 presents the recovery percentage [43], illustrating the reliability and precision of the new technique employed in this research, with percentages approaching as close to 100% as possible. This was achieved by simulating the detection of analytical standard PQ contamination in water samples from various sources at a concentration of 10^−7^ M (or 25.7 µg/L). Specifically, for the detection of PQ in any given water sample, a calibration curve must first be established within that sample matrix. Subsequently, for the measurement of a water sample with an unknown concentration of PQ, the primary peak intensity of the sample can simply be substituted into the equation of the established calibration curve to determine the concentration. Table 5, show comparison of PQ studies on different SERS colloids.

## 4. Conclusions

In this study, we successfully developed a highly efficient alternative method for detecting PQ residues in water. The approach is convenient, cost-effective, and practical. TSNP colloids were simply mixed with PQ-contaminated water at a 1:1 ratio and incubated for 10 min, followed by drop-casting and Raman measurement. Analysis of the EF, SNR, and RSD demonstrated high Raman signal enhancement, excellent stability, and reproducibility. Remarkably, the method enabled PQ detection at concentrations as low as 10^−16^ M, an unprecedented level, attributed to the strong LSPR from the sharp tips and edges of TSNPs, further enhanced by dielectric spacer effects. Furthermore, the method accurately detected PQ in water samples simulating natural contamination without requiring complex sample extraction, achieving a percentage of recovery greater than 90%. The insights gained from this study can be extended to other pesticides with properties similar to PQ. Ultimately, this approach holds promise for real-world applications in the field, in combination with portable Raman spectrometers, enabling organic farmers, governmental and private agencies, or other stakeholders to reduce procedural steps, time, and costs when analyzing large numbers of samples, thereby improving food and beverage safety for consumers.

## Figures and Tables

**Table 1:** Typical vibrational bonds and their associated Raman shift of PQ [23].

Raman Shift (cm^−1^)	Vibrational Bond
841	carbon–nitrogen single bonds (C-N)
1192	carbon–carbon double bonds (C=C)
1298	carbon–carbon single bonds (C-C)
1651–1655	carbon–nitrogen double bonds (C=N)

**Table 2:** SNR and EF values of 10^−4^ M PQ mixed with TSNP colloids for different mixing durations.

Mixing Time (min)	SERS Signal Intensity (a.u.)	SNR	EF
Non-SERS (control)	20.77	3.15	1
5	23,215.80	110.78	117.76
10	52,302.80	255.84	2518.19
15	23,215.80	133.99	1117.76
20	18,845.97	182.62	907.36
30	28,236.47	186.91	1359.48
45	13,623.97	126.70	655.94

**Table 3:** SNR and EF values of 10^−6^–10^−16^ M PQ detection using TSNP colloidal SERS.

Mixing Time (min)	SERS Signal Intensity (a.u.)	EF
blank (pure 10^−4^ M)	21	3
10^−6^	30,775	119
10^−8^	21,117	190
10^−10^	14,460	114
10^−12^	7371	364
10^−13^	2286	65
5 × 10^−14^	2209	12
5 × 10^−16^	708	16
10^−16^	629	43

**Table 4:** Comparing recovery percentage of various water samples which were spiked with 10^−7^ M analytical standard PQ using TSNP colloidal SERS.

Sample	Calibration Curve Equation	TSNP SERS	
		Peak Intensity	Recovery
tap water (Suphanburi)	y = 5575.6x − 3050.4	7516.10	92.78
tap water (Bangkok)	y = 10,491x − 4638.8	15,931.95	97.48
River (Chao Phraya)	y = 3786.7x + 3595	10,742.35	96.19
drinking water	y = 2.8055 × 10^3.7413^	12,841.50	113.58

x is the log concentration (nM), although 10^−7^ M is equal to 10^2^ nM, so x = 2.

**Table 5:** Comparison of PQ studies on different SERS colloids.

SERS Type	Sample	LOD (M)	Ref
Fe_3_O_4_@Ag magnetic NPs	water	10^−10^	[45]
AgNPs on lotus leaf	water	4.8 × 10^−12^	[46]
Au NPs–resin sphere	water	10^−12^	[47]
AuNPs SERS-based aptasensor	water	1.4 × 10^−7^	[48]
AuNPs on 3D structured aluminum sheet	water	10^−7^	[20]
TSNP colloidal SERS	DI water	10^−16^	This work

## Data Availability

The original contributions presented in the study are included in the article, further inquiries can be directed to the corresponding author.
